# Parameterized Verification of Systems with Global Synchronization and Guards

**DOI:** 10.1007/978-3-030-53288-8_15

**Published:** 2020-06-13

**Authors:** Nouraldin Jaber, Swen Jacobs, Christopher Wagner, Milind Kulkarni, Roopsha Samanta

**Affiliations:** 8 Microsoft Research Lab, Redmond, WA USA; 9 University of Southern California, Los Angeles, CA USA; 10 Purdue University, West Lafayette, USA; 11 CISPA Helmholtz Center for Information Security, Saarbrücken, Germany

## Abstract

Inspired by distributed applications that use consensus or other agreement protocols for global coordination, we define a new computational model for parameterized systems that is based on a general global synchronization primitive and allows for global transition guards. Our model generalizes many existing models in the literature, including broadcast protocols and guarded protocols. We show that reachability properties are decidable for systems without guards, and give sufficient conditions under which they remain decidable in the presence of guards. Furthermore, we investigate cutoffs for reachability properties and provide sufficient conditions for small cutoffs in a number of cases that are inspired by our target applications.

## Introduction

Distributed applications are notoriously difficult to implement and reason about, primarily due to the combinatorial explosion of behaviors resulting from the interleaving of computation and communication. Naturally, they have received a lot of attention from the formal methods community to facilitate reasoning about correctness properties that are too complex to reason about informally or manually [3, 7, 14, 15, 34, 36, 42, 46, 50, 52, 55].

One of the main challenges in *fully automated* reasoning about a distributed system is *scalability* in a critical system parameter—the number of processes—with the epitome of success being *parameterized verification of correctness*—correctness that holds regardless of this parameter. Unfortunately, the parameterized verification problem is known to be undecidable even in very simple cases, for example, finite-state processes that pass a 2-valued token in a ring [54]. Hence, approaches for parameterized verification are divided into two groups: (i) ones that support a large class of systems, but only provide semi-decision procedures [1, 41] and (ii) ones that provide fully automatic decision procedures for a well-defined class of systems, but need to carefully restrict this class of systems to obtain such a strong result. While the former cannot provide any guarantee of success, the latter are often not sufficiently general to model practical examples.

In this work, we target fully-automated parameterized verification for a significantly more general class of systems than addressed in prior work (cf. the surveys [9, 21, 26]). Inspired by distributed applications that use consensus or other *agreement protocols* for global coordination, we introduce *global synchronization protocols*, a new computational model for distributed systems that generalizes most of the existing models based on process synchronization, including models based on pairwise rendezvous [32], asynchronous rendezvous [16], negotiation [27] and broadcasts [28]. We show that despite this generality, we can still decide parameterized verification for safety properties. Going beyond that, we show that under certain conditions, our model can be augmented with global transition guards—which allow to model semaphore-based access control as well as preconditions for global consensus-like coordination—while retaining decidability. This makes our model one of the most expressive models for which the parameterized verification problem is still decidable. Furthermore, we present several results on *cutoffs* for our model, i.e., the number of processes sufficient to prove or disprove properties of a parameterized system. Inspired both by the decision procedure and by negative examples that require large cutoffs, we define sufficient conditions on systems in our computational model that make small, practical cutoffs possible. Finally, we evaluate our approach on several distributed applications, showing that they can indeed be modeled as global synchronization protocols, and we illustrate the significance of our cutoff results in the verification of these benchmarks.

**Motivating Example.** Our system model is inspired by applications that *use* agreement protocols, like leader election or consensus, as building blocks to achieve a more complex overall functionality. We are interested in a compositional verification setting where we *assume* that the agreement protocols have been verified separately and want to *guarantee* the overall correctness of an application without having to explicitly model and verify the agreement protocols within the application; in particular, we focus on a setting where verified agreement protocols are encapsulated into an abstraction with precondition obligations and postcondition guarantees.

**Fig. 1.**
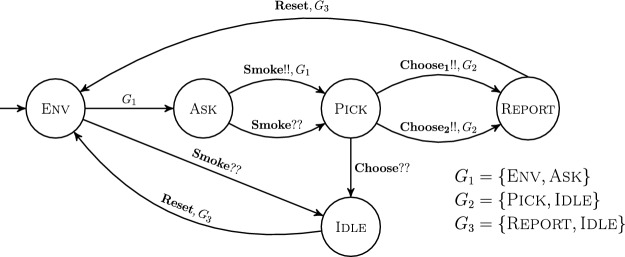
A smoke detector process. The internal transition from initial state Env to Ask models that a process detects smoke (an environment signal). A process that detected smoke can initiate a broadcast $\mathbf {Smoke}$, moving all processes from Env to Idle and from Ask to Pick, where the transition $\mathbf {Choose}$ moves (up to) 2 processes to Report, and the rest from Pick to Idle. Finally, all processes from Report and Idle may move back to Env in a synchronous transition with no dedicated sender. Transitions labeled with a set $G_i$ can only be taken if all processes are in this set. The safety property for a distributed smoke detector based on this process is that at most 2 processes should report the fire.

Thus, our system model needs to be able to incorporate such pre- and postconditions of agreement protocols. As a simple example, consider the smoke detector application in Fig. 1 whose intended behavior is as follows. Upon detecting smoke, the processes coordinate to choose (up to) 2 processes to report the smoke to the fire department. It uses different types of transitions, several of which are popular in the literature and are supported by existing decidability results: an *internal transition* (from state Env to state Ask), a *broadcast* (on action $\mathbf {Smoke}$), and a *negotiation*, i.e., a synchronous transition of all processes with no distinguished sender (on action $\mathbf {Reset}$). However, additionally our application requires that some transitions can only happen under certain conditions, given by guards $G_i$ in transition labels. For example, action $\mathbf {Reset}$ should only be possible if all processes are in $G_3$, i.e., in states Report or Idle. And most importantly, in state Pick we want the system to *agree* on (up to) 2 processes that move into state Report. This requires a novel type of transition that we have not found in existing literature, allowing two processes to take a distinguished role while all other processes are treated uniformly. To faithfully model agreement of processes, we also require a guard on this transition, since any agreement protocol is based on the assumption that all processes are ready (i.e., their local state satisfies some condition) before invocation of the protocol.

## System Model: Global Synchronization Protocols

We present *global synchronization protocols* (GSPs), a formal system model that generalizes most of the existing synchronization-based models in the literature [16, 27, 28, 32], including models based on rendezvous and broadcasts. In this model, each global transition synchronizes all processes, where an arbitrary number *k* of processes *act* as the senders of the transitions, while the remaining processes react uniformly as receivers. The model supports two basic types of transitions: (i) a *k-sender transition*, which *can fire* only if at least *k* processes are ready to act as senders, and *is fired* with exactly *k* processes acting as senders, and (ii) a *k-maximal transition*, which *can fire* if the number *m* of processes that are ready to act as senders is at least 1, and *is fired* with *min*(*m*, *k*) processes acting as senders. Additionally, each transition can be equipped with a *global guard* that identifies a subset of the local state space. Then, a transition is *enabled* whenever it can fire and the local states of all processes are in the set identified by the transition guard.

We formalize these notions in the following, starting with the case without transition guards.

### Global Synchronization Without Guards

**Unguarded Processes.** An *unguarded process* is a labeled transition system $P=\langle A,S,s_0,T \rangle$, where *A* is a set of *local actions*, $S$ is a finite set of states, $s_0 \in S$ is the initial state, and $T \subseteq S\times A \times S$ is the transition relation. *A* is based on a set $\mathcal {A}$ of *global actions*, where each $a \in \mathcal {A}$ has an *arity* $k \ge 1$ and is either a *k-sender action* or a *k-maximal action*. For every global action $a \in \mathcal {A}$ with arity *k*, *A* contains *local actions* $a_1{!!},\ldots ,a_k{!!},a{??}$. Actions $a_1{!!},\ldots ,a_k{!!}$ are called *sending actions* and *a*?? is called a *receiving action*.

A local transition from state $s$ to state $s'$ on sending action $\alpha \in A$ denoted $s \xrightarrow {\alpha } s'$ is called a *sending transition* (resp., *receiving transition*) if $\alpha$ is a sending action (resp., receiving action). We assume that receives are *deterministic*: for each state $s$ and each receiving action *a*??, there is exactly one state $s'$ with $s \xrightarrow {a{??}} s'$, and that sends are *unique*: for each sending action $a_i$ there is exactly one pair of states $s, s'$ with $s \xrightarrow {a_i{!!}} s'$.^1^

#### Example 1

If we ignore guards on transitions, the process in Fig. 1 is an unguarded process. Global action **Choose** has arity 2, and local sending transitions  for $i \in \{1,2\}$. One local receiving transition is , and all other receiving transitions on **Choose** are self-loops (not depicted).

**Unguarded Systems.** Given an unguarded process $P=\langle A,S,s_0,T \rangle$, we consider systems composed of *n* identical processes, and use a counter abstraction to efficiently represent global states, without loss of precision [25].^2^

That is, the parameterized global transition system is defined as $\mathcal {M}(n)=\langle \mathcal {A},Q,\mathbf {q}_0,\rightarrow \rangle$, where $Q = \{0,\ldots ,n\}^{S}$, i.e., a global state is a function $\mathbf {q}: S\rightarrow \{0,\ldots ,n\}$. Assuming a fixed order on $S$, we will also use $\mathbf {q}$ as a vector of natural numbers. The initial state $\mathbf {q}_0$ is the state with $\mathbf {q}_0(s_0)=n$ and $\mathbf {q}_0(s)=0$ for all $s\ne s_0$. Finally, we define the global transition relation $\rightarrow$, separated into the two different types of actions:

*k-sender Actions.* A *k*-sender action $a \in \mathcal {A}$ with local sending transitions $s_i \xrightarrow {a_i{!!}} s_i'$ for $i \in \{1,\ldots ,k\}$ can be fired from a global state $\mathbf {q}$ if there are *k* processes that can take these local transitions. Upon firing the action, each of the local transitions on actions $a_i!!$ is taken by exactly one process, and all other processes take a transition on action *a*?? to arrive in the new global state $\mathbf {q}'$. Formally, we assign to each *k*-sender action $a \in \mathcal {A}$ (i) a vector $\mathbf{v} _a \in Q$ containing the number of expected senders for each state $t \in S$: $\mathbf{v} _a(t)= | \{ s \xrightarrow {a_i{!!}} s' \mid s= t \}|$, (ii) a vector $\mathbf{v} _a'$ containing the number of senders that will be in each state $t \in S$ after the transition: $\mathbf{v} _a'(t)= | \{ s \xrightarrow {a_i{!!}} s' \mid s' = t \}|$, and (iii) a function $M_a: S\times S\rightarrow \{0,1\}$, where $M_a(s,s')=1$ if there is a local transition $s \xrightarrow {a??} s'$, and $M_a(s,s')=0$ otherwise. We also use $M_a$ as a $|S| \times |S|$ matrix, called the *synchronization matrix* of action *a*.

Then, a transition from global state $\mathbf {q}$ on action *a* is possible if $\mathbf {q}(s_i) \ge \mathbf{v} _a(s_i)$ for all $i \in \{1,\ldots ,k\}$, and the resulting global state can be computed as

$$\mathbf {q}' = M_a \cdot (\mathbf {q}- \mathbf{v} _a) + \mathbf{v} _a',$$

and we write $\mathbf {q} \xrightarrow {a} \mathbf {q}'$. Intuitively, $\mathbf {q}'$ is obtained from $\mathbf {q}$ by “removing” the senders from their local start states, moving all the remaining (receiving) processes to their respective local destination states, and then adding the senders to their appropriate local destination states. Note that this representation relies on the assumption that sends are unique and receives are deterministic, which also implies that each column of a synchronization matrix $M_a$ is a unit vector.

#### Example 2

Consider the process in Fig. 1. The synchronization matrix and vectors for action $\mathbf {Smoke}$ are shown below, with global states given in the order $\langle \textsc {Env} ,\ \textsc {Ask}, \ \textsc {Idle}, \ \textsc {Pick}, \ \textsc {Report} \rangle$ (and abbreviated as $\langle \textsc {E} ,\ \textsc {A}, \ \textsc {I}, \ \textsc {P}, \ \textsc {R} \rangle$). Notice, for instance, that the first column in $M_{\mathbf {Smoke}}$ encodes the local receive transition $\textsc {Env} \xrightarrow {\mathbf {Smoke}??} \textsc {Idle}$. The vector-pair $\mathbf{v} _{\mathbf {Smoke}}$ and $\mathbf{v} '_{\mathbf {Smoke}}$ encode the local send transition $\textsc {Ask} \xrightarrow {\mathbf {Smoke}!!} \textsc {Pick}$. In particular, $\mathbf{v} _{\mathbf {Smoke}}$ indicates that the sender starts in Ask and $\mathbf{v} '_{\mathbf {Smoke}}$ indicates that the sender moves to Pick. 
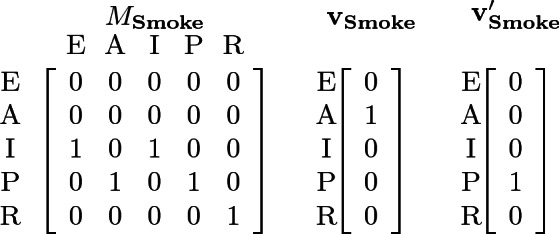


Now, consider a global state $\langle 3, 2, 0, 0, 0 \rangle$ with three processes in Env and two in Ask. From this state, the transition $\langle 3, 2, 0, 0, 0 \rangle \xrightarrow {\mathbf {~Smoke}} \langle 0, 0, 3, 2, 0 \rangle$ is enabled (since there is at least 1 sender in $\textsc {Ask}$), where all three processes in Env act as receivers to move to Idle (according to the synchronization matrix $M_{\mathbf {Smoke}}$), one process in Ask acts as the sender to move to Pick, and the other process in Ask acts as a receiver, also moving to Pick.

*k-maximal Actions.* A *k*-maximal action $a \in \mathcal {A}$ with local sending transitions $s_i \xrightarrow {a_i!!} s_i'$ for $i \in \{1,\ldots ,k\}$ can be fired from a global state $\mathbf {q}$ if there is at least one process that can take one of these local transitions. Upon firing the action, for each state $s_i$ with at least one local transition $s_i \xrightarrow {a_i!!} s_i'$, (i) if $\mathbf {q}(s_i) \ge \mathbf{v} _a(s_i)$ then each of the local transitions $s_i \xrightarrow {a_i!!} s_i'$ is taken by exactly one process, or, (ii) if $\mathbf {q}(s_i) < \mathbf{v} _a(s_i)$ then a total of $\mathbf {q}(s_i)$ of the local transitions $s_i \xrightarrow {a_i!!} s_i'$ are taken, each by exactly one process. All other processes take a transition on the receiving action *a*?? to arrive in the new global state $\mathbf {q}'$. Formally, we again assign to each action *a* vectors $\mathbf{v} _a, \mathbf{v} '_a$ and a synchronization matrix $M_a$, as above. If $\mathbf {q}(s_i) \ge \mathbf{v} _a(s_i)$ for all $i \in \{1,\ldots ,k\}$, then these are used as defined above. For cases where this does not hold, we assign to the action an additional set of vector-pairs $(\mathbf{u} _a,\mathbf{u} '_a)$ with different numbers of senders that actually participate, and $\mathbf {q}'$ is computed based on a vector-pair with the maximal number of senders that is supported by $\mathbf {q}$.

#### Example 3

The synchronization matrix and vectors for action $\mathbf {Choose}$ are shown below. Note that, if $\mathbf {Choose}$ is a 2-maximal action, then the vector-pair $(\mathbf{u} _{\mathbf {Choose}},$ $\mathbf{u} '_{\mathbf {Choose}})$ is used to model the case where only one sender is available to take the sending transition. 
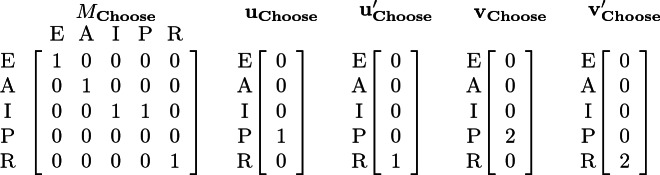


Regardless of whether $\mathbf {Choose}$ is a 2-sender or a 2-maximal action, the global transition $\langle 0, 0, 1, 4, 0 \rangle \xrightarrow {\mathbf {Choose}} \langle 0, 0, 3, 0, 2 \rangle$ is possible. In a state $\mathbf {q}= \langle 0, 0, 4, 1, 0 \rangle$, with 4 processes in Idle and 1 in Pick, the $\mathbf {Choose}$ action will not be enabled if it is a 2-sender action because two sending processes are required (in Pick), but only one sender is available. However, if $\mathbf {Choose}$ is a 2-maximal action, then the global transition $\langle 0, 0, 4, 1, 0 \rangle \xrightarrow {\mathbf {Choose}} \langle 0, 0, 4, 0, 1 \rangle$ is possible.

**Runs, Reachability Properties.** A *run* of system $\mathcal {M}(n)$ is a finite or infinite sequence of global states $\mathbf {q}_0 \mathbf {q}_1 \ldots$, where $\mathbf {q}_0$ is the initial state and $\mathbf {q}_i \xrightarrow {a} \mathbf {q}_{i+1}$ for all *i*. We say that a state $\mathbf {q}$ is *reachable* in $\mathcal {M}(n)$ if there is a run of $\mathcal {M}(n)$ that ends in $\mathbf {q}$. For a fixed $m \in \mathbb {N}$ and local state $s\in S$, let $\phi _m(s)$ be a property denoting the reachability of a global state $\mathbf {q}$ with $\mathbf {q}(s)\ge m$. If such a state is reachable in $\mathcal {M}(n)$, we write $\mathcal {M}(n) \models \phi _m(s)$.

**Other Communication Primitives in the GSP Model.** Note that most of the synchronization-based communication primitives from the literature are instances of *k*-sender transitions or *k*-maximal transitions: *broadcasts* [28] are simply 1-sender transitions, *internal transitions* are 1-sender transitions with $M_a=Id$ (the identity matrix), *pairwise rendezvous transitions* [32] are 2-sender transitions (denoting the sender and receiver of the rendezvous transition) with $M_a=Id$, *asynchronous rendezvous transitions* [16] are 2-maximal transitions with $M_a=Id$. *Negotiations* [27], i.e., a synchronous transition of all processes with no distinguished sender, can be modeled as a set of 1-sender transitions, where every local receiving transition $s \xrightarrow {a??} s'$ is paired with a sending transition $s \xrightarrow {a!!} s'$, allowing an arbitrary process to act as the sender. In addition to these, GSPs allow us to express many other natural synchronization primitives, e.g., summarizing the election of (up to) *k* leaders in a single step.

Finally, *disjunctive guards* [19], i.e., guards $G\subseteq S$ that require that there *exists* a process that is in some state $s\in G$, can be modeled by adding an auxiliary sending action $a_{G}!!$, and transitions $s \xrightarrow {a_{G}!!} M_a( s)$ for every $s\in G$, i.e., a process in some state $s\in G$ must exist to enable the transition, but apart from that this process acts like a receiver. Note that this works without adding a notion of guards to our model.

In what follows, we extend our model to allow *conjunctive guards*, i.e., guards that require that *all* processes are in some subset of the local state space.

### Global Synchronization with Guards

**Guarded Processes.** A *guarded process* is a tuple $P_{GSP}=\langle A,S,s_0,T \rangle$, where all components are as before, except that now we have $T \subseteq S\times A \times \mathcal {P}(S)\times S$, i.e., transitions are additionally labeled with a subset of $S$, called a *guard*. A local transition from state $s$ to state $s'$ on action $\alpha$ with guard $G$ will be denoted $s \xrightarrow {\alpha ,G} s'$. We call a guard $G$ *non-trivial* if $G\ne S$. Wlog, we assume that for any global action *a*, all local transitions based on *a* have the same guard.

**Guarded Systems.** Let the *support* of a global state $\mathbf {q}$ be $\mathsf {supp} (\mathbf {q}) = \{ s\in S\mid \mathbf {q}(s) > 0 \}$, i.e., the set of local states that appear at least once in $\mathbf {q}$. Then the semantics of a global transition on action *a* with guard *G*, denoted $\mathbf {q} \xrightarrow {a, G} \mathbf {q}'$, is as defined before, except that the transition is enabled only if $\mathsf {supp} (\mathbf {q}) \subseteq G$.

#### Example 4

Consider the global transitions introduced in Example 2, and recall that global states are given in the order $\langle \textsc {Env} ,\ \textsc {Ask}, \ \textsc {Idle}, \ \textsc {Pick}, \ \textsc {Report} \rangle$. While the transition $\langle 0, 0, 1, 4, 0 \rangle \xrightarrow {\mathbf {~Reset ~}} \langle 1, 0, 0, 4, 0 \rangle$ would be possible in the unguarded model, the guard $G_3 = \{\textsc {Report},\textsc {Idle}\}$ on the $\mathbf {Reset}$ action disables this transition, as $\mathsf {supp} (\langle 0, 0, 1, 4, 0 \rangle ) = \{\textsc {Pick},\textsc {Idle}\} \not \subseteq G_3$. Similarly, from $\mathbf {q}= \langle 1 , 0 , 1 , 2 ,0 \rangle$, while a transition on action $\mathbf{Choose}$ is enabled for unguarded processes, the guard $G_2=\{\textsc {Pick},\textsc {Idle}\}$ on action $\mathbf {Choose}$ disables this transition, since $\mathsf {supp} (\langle 1 , 0 , 1 , 2 ,0 \rangle ) \not \subseteq G_2$.

## Parameterized Verification for GSPs Without Guards

In this section, instead of the parameterized system $\mathcal {M}(n)$, we consider an infinite-state system $\mathcal {M}_\infty$ that includes the behaviors of $\mathcal {M}(n)$ for every *n*: it initializes to $\mathcal {M}(n)$ for arbitrary $n \in \mathbb {N}$, and then behaves according to the semantics of a GSP of that size. We are interested in reachability properties $\phi _m(s)$, where $\mathcal {M}_\infty \models \phi _m(s)$ is equivalent to $\exists n.~ \mathcal {M}(n) \models \phi _m(s)$, i.e., we are considering a *parameterized reachability property* over all instances of $\mathcal {M}$.

We use this slightly different model in order to make use of the notion of *well-structured transition systems (WSTS)*, as defined by Finkel [30]: an infinite-state transition system that is equipped with a *well-quasi-order (WQO)* on its state space and has some additional properties. Finkel and Schnoebelen [31] have surveyed existing results on WSTSs and put them into a common framework.

We will show that, for a suitable WQO, $\mathcal {M}_\infty$ is a WSTS, and that this enables parameterized verification for reachability properties $\phi _m(s)$.

### Compatibility and Effective Computability of Predecessors

For the following definitions, fix an infinite set of states $Q$ and a transition relation $\rightarrow$. Moreover, let $\preceq$ be a WQO on $Q$, i.e., a reflexive and transitive relation such that, for any infinite sequence $\mathbf {q}_0, \mathbf {q}_1, \mathbf {q}_2, \ldots$ of states from $Q$, there exist indices $i < j$ with $\mathbf {q}_i \preceq \mathbf {q}_j$. In particular, $\preceq$ does not admit infinitely decreasing sequences or infinite anti-chains.

**Compatibility.** We say that $\preceq$ is *compatible* with $\rightarrow$ if for every $\mathbf {q}, \mathbf {q}', \mathbf {p}\in Q$ with $\mathbf {q}\preceq \mathbf {p}$ and $\mathbf {q}\rightarrow \mathbf {q}'$ there exists $\mathbf {p}' \in Q$ with $\mathbf {q}' \preceq \mathbf {p}'$ and $\mathbf {p}\rightarrow ^* \mathbf {p}'$. If the property also holds after replacing $\mathbf {p}\rightarrow ^* \mathbf {p}'$ with $\mathbf {p}\rightarrow \mathbf {p}'$, then we say $\preceq$ is *strongly compatible* with $\rightarrow$.

**Well-Structured Transition System.** A transition system $(Q,\rightarrow )$ equipped with a WQO that is compatible with $\rightarrow$ is called a *well-structured transition system (WSTS)*.

**Upwards-Closed Sets.** For a (possibly infinite) subset $U \subseteq Q$, the *upwards closure* of *U* is the set $\uparrow U = \{ \mathbf {p}\in Q\mid \exists \mathbf {q}\in U: \mathbf {q}\preceq \mathbf {p}\}$. A set *U* is *upwards closed* if $\uparrow U = U$. Every upwards closed set *U* has a finite *basis*: a finite set $B \subseteq U$ such that $\uparrow B = U$.

**Effectively Computable Predecessors.** For $U \subseteq Q$, let *Pred*(*U*) denote the predecessor states of *U* with respect to $\rightarrow$. We say that we can *effectively compute Pred* if there exists an algorithm that computes a finite basis of *Pred*(*U*) from any finite basis of any upwards-closed $U \subseteq Q$.

#### Theorem 1

**(**[31]**).** In a WSTS with effectively computable *Pred*, reachability of any upwards-closed set is decidable.

### Decidability for Unguarded GSPs

We prove that any unguarded GSP is a WSTS with effectively computable *Pred*, which implies that reachability properties are decidable for GSPs. To this end, let $\preceq$ be the component-wise order on global state vectors $\mathbf {q}$, $\mathbf {p}$:

$$\mathbf {q}\preceq \mathbf {p}\; \text { iff } \; \mathbf {q}(s) \le \mathbf {p}(s) \text { for all } s\in S.$$

Note that with respect to this WQO, the set of global states $\mathbf {q}$ with $\mathbf {q}(s) \ge m$ is an upwards-closed set, i.e., if we can decide reachability of upwards-closed sets, then we can decide reachability properties $\phi _m(s)$. Thus, decidability of checking $\mathcal {M}_\infty \models \phi _m(s)$ follows from the following theorem.

#### Theorem 2

If $\mathcal {M}_\infty$ is based on an unguarded GSP process, then $\mathcal {M}_\infty$ equipped with $\preceq$ is a WSTS and we can effectively compute Pred.

#### Proof

To prove that $\mathcal {M}_\infty$ is a WSTS, we show strong compatibility of transitions w.r.t. $\preceq$. We consider the following two cases separately: (i) *k*-sender transitions, and (ii) *k*-maximal transitions.

(i) For *k*-sender transitions, let $\mathbf {q}\preceq \mathbf {p}$ and $\mathbf {q} \xrightarrow {a} \mathbf {q}'$ for some *k*-sender action *a*. Then $\mathbf {q}' = M_a \cdot (\mathbf {q}- \mathbf{v} _a) + \mathbf{v} _a'$ for some synchronization matrix $M_a$ and vectors $\mathbf{v} _a, \mathbf{v} _a'$ associated with action *a*. First observe that since $\mathbf {q}\preceq \mathbf {p}$, there is also a transition $\mathbf {p} \xrightarrow {a} \mathbf {p}'= M_a \cdot (\mathbf {p}- \mathbf{v} _a) + \mathbf{v} _a'$. Moreover, we have $M_a \cdot \mathbf {q}\preceq M_a \cdot \mathbf {p}$, and therefore $M_a \cdot (\mathbf {q}- \mathbf{v} _a) + \mathbf{v} _a' \preceq M_a \cdot (\mathbf {p}- \mathbf{v} _a) + \mathbf{v} _a'$, i.e., $\mathbf {q}' \preceq \mathbf {p}'$.

(ii) For *k*-maximal transitions, consider again $\mathbf {q}\preceq \mathbf {p}$ and $\mathbf {q} \xrightarrow {a} \mathbf {q}'$, where now *a* is a *k*-maximal action. Then $\mathbf {q}' = M_a \cdot (\mathbf {q}- \mathbf{u} _{a,\mathbf {q}}) + \mathbf{u} _{a,\mathbf {q}}'$ for some vectors $\mathbf{u} _{a,\mathbf {q}},\mathbf{u} _{a,\mathbf {q}}'$ with $\sum _{s \in S}\mathbf {u}_{a,\mathbf {q}}(s)=\sum _{s \in S}\mathbf {u}_{a,\mathbf {q}}'(s) \le k$. Again, first observe that since $\mathbf {q}\preceq \mathbf {p}$, a transition $\mathbf {p} \xrightarrow {a} \mathbf {p}'$ is enabled, where $\mathbf {p}' = M_a \cdot (\mathbf {p}- \mathbf{u} _{a,\mathbf {p}}) + \mathbf{u} _{a,\mathbf {p}}'$ and $\mathbf{u} _{a,\mathbf {p}}(s) \ge \mathbf{u} _{a,\mathbf {q}}(s)$, $\mathbf{u} _{a,\mathbf {p}}'(s) \ge \mathbf{u} _{a,\mathbf {q}}'(s)$ for all $s\in S$. Note that, for any $s\in S$, we can have $\mathbf{u} _{a,\mathbf {p}}(s) > \mathbf{u} _{a,\mathbf {q}}(s)$ only if $\mathbf {q}(s)-\mathbf{u} _{a,\mathbf {q}}(s) \le 0$ and $\mathbf {p}(s) > \mathbf {q}(s)$. Furthermore, $\mathbf{u} _{a,\mathbf {p}}(s) - \mathbf{u} _{a,\mathbf {q}}(s) \le \mathbf {p}(s) - \mathbf {q}(s)$. Therefore, we get $\mathbf {q}- \mathbf{u} _{a,\mathbf {q}} \preceq \mathbf {p}- \mathbf{u} _{a,\mathbf {p}}$, which implies $M_a \cdot (\mathbf {q}- \mathbf{u} _{a,\mathbf {q}}) \preceq M_a \cdot (\mathbf {p}- \mathbf{u} _{a,\mathbf {p}})$, and thus $M_a \cdot (\mathbf {q}- \mathbf{u} _{a,\mathbf {q}}) + \mathbf{u} _{a,\mathbf {q}}' \preceq M_a \cdot (\mathbf {p}- \mathbf{u} _{a,\mathbf {p}}) + \mathbf{u} _{a,\mathbf {p}}'$, i.e., $\mathbf {q}' \preceq \mathbf {p}'$.

Next, we prove that we can effectively compute the basis of *Pred*(*C*), where *Pred*(*C*) is the set of states from which a transition exists to a state in an upwards-closed set *C*, as follows:

(i) For a *k*-sender transition based on action *a*, any predecessor $\mathbf {q}$ in *Pred*(*C*) must satisfy (i) $\mathbf{v} _a \preceq \mathbf {q}$, and (ii) $M_a \cdot (\mathbf {q}- \mathbf {v}_a) + \mathbf{v} _a' = \mathbf {q}'$, for some $\mathbf {q}' \in C$. The basis of *Pred*(*C*) consists of the minimal elements (w.r.t. $\preceq$) that satisfy these conditions, and thus is computable.

(ii) For *k*-maximal transitions, the proof works in the same way, except that now we may have multiple possibilities of what a minimal predecessor could be, based on different subsets of the senders being present or not. Since this is always a finite case distinction, effective computability of *Pred* is still guaranteed.    $\square$

## Parameterized Verification for GSPs with Guards

For GSPs with guards, compatibility under $\preceq$ in general does not hold, since for $\mathbf {q}\preceq \mathbf {p}$, a transition on action *a* that is enabled in $\mathbf {q}$ may not be enabled in $\mathbf {p}$. Furthermore, note that even strong restrictions on processes are unlikely to yield compatibility with respect to $\preceq$, since whenever $\mathsf {supp} (\mathbf {q})\subseteq G$ for a non-trivial $G$, one can always find a $\mathbf {p}$ with $\mathbf {q}\preceq \mathbf {p}$ and $\mathsf {supp} (\mathbf {p}) \nsubseteq G$, disabling the action.

Therefore, we introduce a refined WQO, denoted $\trianglelefteq$, that is based on the semantics of guards, as well as sufficient conditions on the guarded process *P*, such that the system $\mathcal {M}_\infty$ is a WSTS and we can effectively compute *Pred*.

Let $\mathcal {G}$ be the set of guards that appear on transitions in *P*, and recall that $\mathsf {supp} (\mathbf {q}) = \{ s\in S\mid \mathbf {q}(s) > 0 \}$. Then we consider the following WQO^3^:

$$\mathbf {q}\trianglelefteq \mathbf {p}\; \text { iff } \; \left( \mathbf {q}\preceq \mathbf {p}\wedge \forall G\in \mathcal {G}: \left( \mathsf {supp} (\mathbf {q}) \subseteq G\iff \mathsf {supp} (\mathbf {p}) \subseteq G\right) \right) .$$

Intuitively, a global state $\mathbf {p}$ is considered greater than a global state $\mathbf {q}$ if $\mathbf {p}$ has at least as many processes as $\mathbf {q}$ in any given state, *and* for every transition $\mathbf {q} \xrightarrow {a} \mathbf {q}'$ that is enabled in $\mathbf {q}$, a transition on action *a* is also enabled in $\mathbf {p}$.

We will see that compatibility with respect to $\trianglelefteq$ can only be ensured under additional conditions, as formalized in the following.

### Guard-Compatibility and Well-Behaved Processes

**Strong Guard-Compatibility for** ***k*-Sender Actions.** For a *k*-sender action *a* with local sending transitions $s_i \xrightarrow {a_i!!, G} s_i'$ for $i \in \{1,\ldots ,k\}$, let $\hat{s}$ be the set of all states $s_i$, $\hat{s} '$ the set of states $s_i'$, and $M_a$ the synchronization matrix. We say that action *a* is *strongly guard-compatible* if the following holds for all $G' \in \mathcal {G}{ :}$

C1 $$\begin{aligned} \hat{s} ' \subseteq G' \Rightarrow \forall s\in G{:}~ M_a(s) \in G' \end{aligned}$$

Intuitively, if all senders move into a guard $G'$, then also all receivers need to move into $G'$. This ensures that if $G'$ is satisfied after the transition in a system of a given size, then it is satisfied after that transition in a system of any bigger size, because any additional receivers must also move into $G'$. Note that Condition (C1) always holds for trivial guards.

**Strong Guard-Compatibility for k-Maximal Actions.** For a *k*-maximal action *a*, the idea of the condition is the same as before, but it must be extended to allow different subsets of the *potential* senders to act as *actual* senders in a given transition with action *a*. A simple approximation is that all senders must agree, for every $G\in \mathcal {G}$, on whether they enter $G$ or not.

In the following, we formalize a notion that takes into account that transitions that only use a subset of the potential senders are only possible from certain global states, and that global states with different sets of actual senders may be incomparable with respect to $\trianglelefteq$, and therefore unproblematic for compatibility.

We write $t \triangleleft s$ if, for all guards $G\in \mathcal {G}$, $s \in G\Rightarrow t \in G$. Similarly, we write $t \triangleleft H$ for a set of states *H* if, for all guards $G\in \mathcal {G}$, $H \subseteq G\Rightarrow t \in G$.

Consider a *k*-maximal action *a* with local transitions $s_i \xrightarrow {a_i!!,G } s_i'$ for $i \in \{1,\ldots ,k\}$ and synchronization matrix $M_a$. Let $R = G \setminus \{s_1,\ldots ,s_k\}$ and let $\mathcal {G}'$ be the set of all guards $G_R \in \mathcal {G}$ such that $R \subseteq G_R$.

Then we say the action *a* is *strongly guard-compatible* if both of the following hold for all $G' \in \mathcal {G}{ :}$

C2.1 $$\begin{aligned} \left( \bigvee _{1 \le i \le k} s_i' \in G' \right) \Rightarrow \left( \forall s\in R: M_a(s) \in G' \right) \end{aligned}$$

C2.2 $$\begin{aligned} \bigwedge _{i,j \in \{1,\ldots ,k\}} \left( (s_i \triangleleft s_j \wedge s_j' \in G') \Rightarrow (s_i' \in G' \wedge M_a(s_i) \in G') \right) \end{aligned}$$

Intuitively, if one potential sender moves from a state $s_j$ into a guard $G'$, then every receiver from *R* must do the same, so that $G'$ will be satisfied regardless of the number of receivers. This is also required for other senders and receivers from a state $s_i \notin R$, unless there exists a guard that is satisfied if $s_j$ is occupied, but not if $s_i$ is occupied, since that means that a global state where only $s_j$ is occupied is incomparable (w.r.t. $\trianglelefteq$) to a state where also $s_i$ is occupied, and therefore we do not care about compatibility of the transitions.

Note that for $k=1$, the first condition (C2.1) instantiates to condition (C1) and the second condition (C2.2) is an empty conjunction, i.e., vacuously satisfied. This is to be expected, since semantically there is no difference between a 1-sender action and a 1-maximal action.

#### Example 5

We can see that actions $\mathbf {Smoke}$, $\mathbf {Choose}$, and $\mathbf {Reset}$ from our motivating example in Fig. 1 are strongly guard-compatible:

- $\mathbf {Smoke}$ is a 1-sender action with sending transition $\textsc {Ask} \xrightarrow {\mathbf {Smoke}!!, \{\textsc {Env, Ask}\}} \textsc {Pick}$. The state Pick is only included in one non-trivial guard $G_2$ = {Pick, Idle}. Since receiving transitions from {Env, Ask} end in $\{\textsc {Pick, Idle}\} \subseteq G_2$, condition (C1) holds, so $\mathbf {Smoke}$ is strongly guard-compatible.
- Consider $\mathbf {Choose}$ with sending transitions $\textsc {Pick} \xrightarrow {\mathbf {Choose _i!!},\{\textsc {Pick,Idle}\}} \textsc {Report}$ for $i \in \{1,2\}$ as a 2-sender action. Report is only included in one non-trivial guard $G_3$ = {Report, Idle}. Since the receiving transition from {Pick} ends in $\textsc {Idle} \in G_3$ as well, (C1) holds, so $\mathbf {Choose}$ is strongly guard-compatible.
- Consider $\mathbf {Choose}$ as a 2-maximal action. Again, Report is only included in one non-trivial guard $G_3$ = {Report, Idle}. Since all senders and receivers start from Pick and end up in a state in $G_3$, conditions (C2.1) and (C2.2) hold and $\mathbf {Choose}$ is, again, strongly guard-compatible.
- $\mathbf {Reset}$ is a negotiation action. Recall that negotiations are modeled as a set of 1-sender actions, allowing for an arbitrary sender. Therefore, each of these broadcasts must satisfy (C1) for the negotiation to be guard-compatible. $\mathbf {Reset}$ is indeed strongly guard-compatible because all of its sending and receiving transitions end in Env, meaning that when the action fires, all processes will move into a single state, ensuring that all guards will be uniformly enabled or disabled, regardless of the number of processes, which of them is the sender, or whether they begin in Report or Idle.
- Finally, as stated in Sect. 2.1, the internal transition $\textsc {Env} \xrightarrow {G_1} \textsc {Ask}$ can be modeled by a 1-sender action, say *a*, with a send transition $\textsc {Env} \xrightarrow {a!!,G_1} \textsc {Ask}$ and self-loop receive transitions on all states. The sender ends up in one non-trivial guard $G_1=\{\textsc {Env},\textsc {Ask}\}$. Since receiving transitions from $\{\textsc {Env, Ask}\}$ end in $\{\textsc {Env, Ask}\} \subseteq G_1$, condition (C1) holds, so *a* is strongly guard-compatible.

**Refinement: Weak Guard-Compatibility.** To support a larger class of systems, we show how one can relax the previous conditions, at the cost of making them more complex. The idea is that, instead of requiring that if the sender ends up in a guard then the receivers *immediately* end up in that guard after the transition, it is enough if the receivers *have a path* to a state in that guard. To avoid unnecessary complexity, we only consider paths of internal transitions.

If there exists a path of unguarded internal transitions from $s$ to $s'$, we write . Then, condition (C1) can be relaxed to

**Figure:**
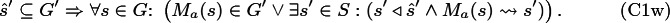

Actions that satisfy condition (C1w) are called *weakly guard-compatible*.

**Remark.** In a similar way, we can relax conditions (C2.1) and (C2.2). Furthermore, the path  of internal transitions can be guarded, as long as the guards are sufficiently general to guarantee that these transitions can be taken. We refer the interested reader to the extended version [38] for more details.

**Well-Behavedness.** Based on guard-compatibility, we can now define the class of processes that will allow us to retain decidability of reachability properties in the parameterized system: We say that a process *P* is **well-behaved** if every action is (weakly) guard-compatible.

Note that unguarded processes are trivially well-behaved.

#### Example 6

Observing that all actions in the process depicted in Fig. 1 are (strongly) guard-compatible, it is clear that the process is **well-behaved**.

**Well-Behaved Systems in the Literature.** We want to point out that many systems studied in the literature are naturally well-behaved.

For example, Emerson and Kahlon [20] introduce a model for cache coherence protocols that is based on broadcast communication and guards. They show that many textbook protocols can be modeled under the following restrictions: (i) every state is assumed to have an unguarded internal transition to the initial state $\textsc {Init}$, and (ii) the only conjunctive guard is $\{ \textsc {Init} \}$. Clearly, every action in a process that satisfies these conditions will also satisfy condition (C1w), and therefore well-behaved systems subsume and significantly generalize the types of protocols considered by Emerson and Kahlon.

Moreover, there has recently been much research on the verification of round-based distributed systems [14, 34, 37], where processes can move independently to some extent, with the restriction that transitions between rounds can only be done synchronously for all processes. When abstracting from certain features (e.g. fault-tolerance and process IDs), our model is well-suited to express such systems: guards can be used to restrict transitions to happen only in a certain round, and can furthermore model the “border” of a round that needs to be reached by all processes, such that they can jointly move to the next round.

Our example from Fig. 1 can also be seen as a round-based system: the first round includes states $\textsc {Env, Ask}$, and upon taking the transition on $\mathbf {Smoke}$, all processes move to the second round, which includes states $\textsc {Pick, Idle}$. From there, on action $\mathbf {Choose}$ the system moves to the third round, which includes states $\textsc {Report, Idle}$, and on action $\mathbf {Reset}$ back to the first round. Note that the states in different rounds are exactly the guards that are used in the transitions—or seen the other way around, guards induce a set of rounds on the local state space, and the guard-compatibility conditions ensure that processes move between these rounds in a systematic way.

While the rounds are very simple in this example, the technique is much more general and can be used to express many round-based systems, including those described in Sect. 6.

### Decidability for Well-Behaved Guarded Processes

Based on the notion of well-behavedness, we can now obtain a decidability result that works in the presence of guards. The following theorem implies that parameterized verification for properties $\phi _m(s)$ is decidable for well-behaved processes.

#### Theorem 3

If $\mathcal {M}_\infty$ is based on a well-behaved GSP process, then $\mathcal {M}_\infty$ is a WSTS and we can effectively compute Pred.

#### Proof

To prove that $\mathcal {M}_\infty$ is a WSTS, we show compatibility of transitions w.r.t. $\trianglelefteq$, i.e., if $\mathbf {q}\trianglelefteq \mathbf {p}$ and $\mathbf {q}\rightarrow \mathbf {q}'$, then $\exists \mathbf {p}'$ with $\mathbf {q}' \trianglelefteq \mathbf {p}'$ and $\mathbf {p}\rightarrow ^* \mathbf {p}'$. We consider two cases: (i) *k*-sender transitions, and (ii) *k*-maximal transitions.

(i) Suppose *a* is a *k*-sender action. Let $\mathbf {q} \xrightarrow {a, G} \mathbf {q}'$ be a transition and $\mathbf {q}\trianglelefteq \mathbf {p}$. Since $\mathbf {q}\trianglelefteq \mathbf {p}$ implies that $\mathsf {supp} (\mathbf {p}) \subseteq G$, we know that transition $\mathbf {p} \xrightarrow {a, G} \mathbf {p}'$ is possible, and by the proof of Theorem 2 we know that $\mathbf {q}' \preceq \mathbf {p}'$. To prove compatibility with respect to $\trianglelefteq$, it remains to show that $\forall G' \in \mathcal {G}: (\mathsf {supp} (\mathbf {q}')\subseteq G' \Rightarrow \mathsf {supp} (\mathbf {p}')\subseteq G')$.

First assume that condition (C1) holds. Then, let $$G' \in \mathcal {G}$$ be an arbitrary guard. By (C1), we either have $$\hat{s} \not \subseteq G'$$, in which case the desired condition is satisfied for $$G'$$, or we have that $$\forall s\in G{:}~ M_a(s) \in G'$$, i.e., all potential receivers move into $$ G'$$. Thus, we get $$\mathsf {supp} (\mathbf {q}')\subseteq G'$$ iff $$\mathsf {supp} (\mathbf {p}') \subseteq G'$$, satisfying the desired condition.

If instead of (C1) the action satisfies (C1w), the argument is the same, except that if necessary we use the internal transitions that are guaranteed to exist by the condition to arrive in a state $\mathbf {p}'$ with $\mathbf {q}' \preceq \mathbf {p}'$.

(ii) Suppose *a* is a *k*-maximal action with local transitions $s_i \xrightarrow {a_i!!,G} s_i'$ for $i \in \{1,\ldots ,k\}$ and synchronization matrix $M_a$. By the proof of Theorem 2 we know that there exists a transition $\mathbf {p} \xrightarrow {a, G} \mathbf {p}'$ with $\mathbf {q}' \preceq \mathbf {p}'$, and it remains to show that $\forall G' \in \mathcal {G}: (\mathsf {supp} (\mathbf {q}')\subseteq G' \iff \mathsf {supp} (\mathbf {p}')\subseteq G')$.

Let $G' \in \mathcal {G}$ be an arbitrary guard, and assume the action is strongly guard-compatible. By condition (C2.1) we know that if there is a single local sending transition with $s_i' \in G'$, then all receivers will move into $G'$. So first suppose there is no such local transition: then $G'$ cannot be satisfied in $\mathbf {q}'$ (since at least one sender must be present), and the desired property holds. Inversely, suppose there is such a local transition: then all processes that start in *R* will be mapped into $G'$, so $G'$ will be satisfied iff all remaining processes are mapped into $G'$. Now, suppose that *all* local transitions taken in $\mathbf {q} \xrightarrow {a, G} \mathbf {q}'$ are such that $s_i' \in G'$ (for otherwise $\mathbf {q}'$ does not satisfy $G'$). Since $\mathbf {q}\preceq \mathbf {p}$, there exists a transition $\mathbf {p} \xrightarrow {a, G} \mathbf {p}'$ such that the set of local transitions that are fired in $\mathbf {q} \xrightarrow {a, G} \mathbf {q}'$ is a subset of the local transitions that are fired in $\mathbf {p} \xrightarrow {a, G} \mathbf {p}'$. If all sending transitions taken in $\mathbf {p} \xrightarrow {a, G} \mathbf {p}'$ are also such that $s_i' \in G'$, then by conditions (C2.1) and (C2.2) the same will hold for all receiving transitions from $\mathbf {p}$, and therefore, $\mathsf {supp} (\mathbf {p}') \subseteq G'$. Thus, suppose there is a local transition $s_i \xrightarrow {a_i!!,G} s_i'$ that is taken in $\mathbf {p} \xrightarrow {a, G} \mathbf {p}'$, but not in $\mathbf {q} \xrightarrow {a, G} \mathbf {q}'$, and $s_i' \notin G'$. Let $s_j \xrightarrow {a_j!!,G} s_j'$ be an arbitrary local transition that is taken in $\mathbf {q} \xrightarrow {a, G} \mathbf {q}'$. Then by condition (C2.2), either there must be a guard $G'' \in \mathcal {G}'$ with $s_i \notin G'' \wedge s_j \in G''$, contradicting the assumption that $\mathbf {q}\trianglelefteq \mathbf {p}$, or we have $s_j' \in G' \Rightarrow s_i' \in G' \wedge M_a(s_i) \in G'$, contradicting the assumption that $s_ i' \notin G'$.

Again, if the action is weakly guard-compatible, the argument can be extended by using the paths of internal transitions, if necessary.

Effective computability of *Pred* follows from the proof of Theorem 2—the only difference is that we must consider the guards, i.e., a predecessor is only valid if it additionally satisfies the guard of the transition under consideration.    $\square$

## Cutoffs for GSPs

We investigate cutoff results for GSPs and their connection to the decidability results in Theorem 2 and 3. While the proofs of these theorems yield a decision procedure for parameterized verification, a cutoff result is more versatile as it reduces parameterized verification to a problem over a fixed number of processes, and under certain conditions can also be used for parameterized synthesis [39].

### Definition and Basic Observations

A *cutoff* for a class of processes $\varPi$ and a class of properties $\varPhi$ is a number $c \in \mathbb {N}$ such that for every $P \in \varPi$ and $\phi \in \varPhi$,

$$ \mathcal {M}_\infty \models \phi \Leftrightarrow \mathcal {M}(c) \models \phi $$

We show how to obtain cutoffs for well-behaved GSPs that satisfy additional conditions, and for reachability properties of the form $\phi _m(s)$, based on observations from the proof of Theorem 2. While for any given parametrized system and any safety property a cutoff exists [45], a general cutoff, even if it can be computed, may be too large to be of practical value: it has been shown that for broadcast protocols the time complexity of checking reachability is non-primitive recursive in the size of the processes [51], and from the proof one can conclude that the same must hold for the size of cutoffs.

**Fig. 2.**
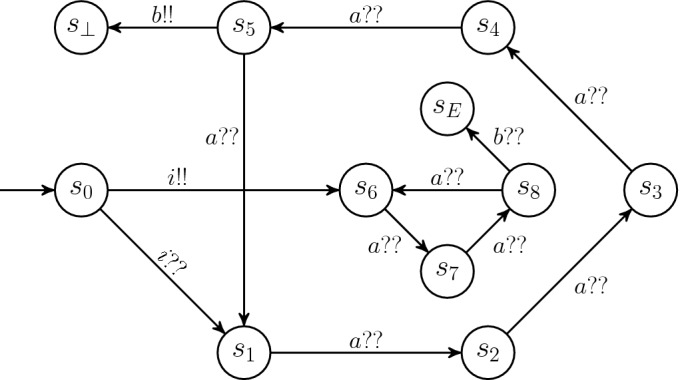
Example witnessing quadratic cutoff. Not depicted are additional sending transitions on *a*!! from every state in the outer cycle to $s_\bot$.

**Example: Quadratic Cutoffs.** Consider the (unguarded) process in Fig. 2. We are interested in a lower bound on the cutoff for this process, with respect to $\phi _1(s_E)$, i.e., reachability of $s_E$ by at least one process. Note that to reach $s_E$, we need at least one process in $s_8$ and one in $s_5$ at the same time. From the initial state $s_0$, the only possible action is *i*, sending one process to $s_6$ in the inner cycle and all other processes to $s_1$ in the outer cycle. Then, the only way to make progress is action *a*, moving the process in the inner cycle to $s_7$, the sending process from $s_1$ to $s_\bot$ (sending transitions on *a*!! are not depicted in Fig. 2), and all other processes to $s_2$. After three further transitions on *a*, the outer processes are in $s_5$, where the sending transition on *b*!! could be fired, but the process in the inner cycle is in $s_7$, so additional transitions on *a* are required. Only after two additional rounds around the outer cycle we arrive in a state where both $s_5$ and $s_8$ are occupied, and we can take the final transition on *b* that takes one process into $s_E$. To arrive there, we took 16 transitions (one on *i*, 14 on *a*, and one on *b*), and by construction every process can only take one sending transition in a run. Thus, we need a system with at least 16 processes to have one of them reach $s_E$, and no smaller number can be a cutoff for $\phi _1(s_E)$.

To see that cutoffs grow at least quadratically, note that in similar examples where the inner and outer cycles consist of $p_1$ and $p_2$ states, respectively, and $p_1$ and $p_2$ are relatively prime, then we need $p_1 \cdot p_2 + 1$ processes to reach $s_E$.

### Conditions for Small Cutoffs

We introduce sufficient conditions on processes that allow us to obtain small cutoffs. These conditions are inspired by our intended applications (see Sect. 6), and based on insights from the decision procedure in the proof of Theorem 2 and the example above. We observe that any $\mathbf {q}\in Pred(C)$ that reaches a state $\mathbf {q}' \in C$ through a *k*-sender action *a* must satisfy (i) $\mathbf{v} _a \preceq \mathbf {q}$, and (ii) $M_a \cdot (\mathbf {q}- \mathbf {v}_a) + \mathbf{v} _a' = \mathbf {q}'$. Thus, if there is $\mathbf {q}' \in C$ such that $\lnot (\mathbf{v} _a \preceq \mathbf {q})$, we need to consider a predecessor $\mathbf {q}$ with $|\mathbf {q}| > |\mathbf {q}'|$. It is easy to see that this can only happen if $\mathbf {q}'$ contains processes in states that can be reached through *a* only through either a receiving transition, or a sending transition if $k>1$. Thus, we want to avoid that states we are interested in are only reachable through such transitions.

We restrict our attention to specifications $\phi _m(s)$ and to cases where we can identify conditions on a GSP process *P* such that the cutoff for such specifications is $c=m$. If this is the case, then we say that reachability of $s$ is *synchronization-independent* in *P*, and that the pair $\langle P,\phi _m(s) \rangle$ is **cutoff-amenable**.

We begin with a simple case, where systems are restricted to only internal transitions and negotiations (we defined in Sect. 2.1 how these are expressed in terms of 1-sender transitions).

#### Lemma 1

Let $P=\langle A,S,s_0,T \rangle$ be a **well-behaved** GSP process such that all transitions are internal transitions or negotiations. Then reachability of *s* is synchronization-independent in *P* for every $s\in S$.

#### Proof

To see this, first consider a system with $n>m$ processes, where eventually *m* of them reach $s$. We can simulate this run in a system with *m* processes by simply keeping the *m* processes that reach $s$, and removing all others. Similarly, if all processes in a system of size *m* eventually reach $s$, then we can simulate this run in a bigger system by adding processes that “follow” the internal transitions of the other processes such that always the same guards as in the original run will be satisfied. Well-behavedness ensures that this is always possible.    $\square$

While we are in general not interested in systems that *only* communicate through internal transitions and negotiations, we can refine this observation based on the states we are interested in, and allow other types of communication.

To this end, define a transition of a process *P* to be *free* if it is (i) an internal transition, (ii) a sending transition of either a broadcast (i.e., a 1-sender action) or a *k*-maximal action, or (iii) a receiving transition $s \xrightarrow {a??,G} s'$ of a broadcast with matching sending transition $s \xrightarrow {a!!,G} s'$. Note that the latter includes negotiation transitions. A path from one state to another is *free* if all transitions on the path are free. The idea is that free transitions and paths are only restricted by guards (i.e., the *absence* of processes in certain states), but not by the *existence* of other processes in certain states (as, e.g., a 2-sender transition would be, since a sender depends on the presence of another sender to be able to fire the global transition and move along its own local transition).

#### Lemma 2

Let $P=\langle A,S,s_0,T \rangle$ be a **well-behaved** GSP process, and $s\in S$ such that **all** paths from $s_0$ to $s$ in *P* are free. Then reachability of *s* is synchronization-independent in *P*.

#### Proof

The argument follows the same line as the one above for protocols with only internal transitions and negotiations, since the same transitions for existing processes are also possible if we can ensure that the same guards can be satisfied in the bigger system. Well-behavedness ensures that there is a run in the bigger system where the same guards are satisfied.    $\square$

We require that all paths be free, since *existence* of a free path is not sufficient in general: if $m>1$, then the first process that moves along that free path may force other processes to leave it (e.g., by taking a sending transition of a broadcast). However, this condition is still slightly restrictive, and can be relaxed.

Define a *simple* path as a path with no repeated states. We show that under additional conditions, it is enough to consider restrictions that are based on paths that are simple *and* free:

#### Lemma 3

Let $P=\langle A,S,s_0,T \rangle$ be a **well-behaved** GSP process, $s\in S$, and let $\mathcal {F}$ be the set of simple free paths from $s_0$ to $s$. If for each send transition:

1. the transition does not appear in paths in $\mathcal {F}$ and the corresponding receiving transitions $s_s \xrightarrow {a??,G_a} s_d$ with $s_s \in p$ for some $p \in \mathcal {F}$ have $s_d=s_s$, or,
2. the transition appears in paths in $\mathcal {F}$ and the following holds for every corresponding receive transition $s_s \xrightarrow {a??,G_a} s_d$ where $s_s \in p$ for some $p \in \mathcal {F}$ and $s_d \notin p$ for any $p \in \mathcal {F}$: either (a) there exists an internal transition $s_s \xrightarrow {} s_d'$ with $s_d' \in p$ for some $p \in \mathcal {F}$, or (b) all paths out of $s_d$ lead back to a state $s_f$ in a path in $\mathcal {F}$ and are free between $s_d$ and $s_f$.

then reachability of $s$ is synchronization-independent in *P*.

#### Proof

First consider a run of a system that satisfies the above conditions, and has $n>m$ processes, where eventually *m* of them reach $s$. We can simulate this run in a system with *m* processes by keeping the *m* processes that reach $s$, and removing all others. Note that the sending transitions are on the same free simple path from which processes can diverge using the corresponding receiving or sending transitions, or they do not affect them at all. Hence, at least one of the senders is guaranteed to reach $s$. All other senders and receivers may diverge from a simple free path but are guaranteed a free path back to a state along a free path and hence, can reach $s$ freely.

Now assume that all processes in a system of size *m* eventually reach $s$, then we can simulate this run in a bigger system by adding processes (that behave in the same way as an existing process). Note that, since any transition diverging from a free simple path can *only* be triggered by a sending transition on that same free path, it is impossible to add a sender that can make processes diverge and then not reach $s$ after.    $\square$

#### Example 7

In this example we show how Lemma 3 applies to the example in Fig. 1. Here $s_0$ is the Env state, $s$ is the Report state, and the value of *m* is 3 (since the safety specification is: no more than 2 detectors can report the fire).

The set of simple free paths $\mathcal {F}$ is:

- $$\textsc {Env} \xrightarrow {} \textsc {Ask} \xrightarrow {\mathbf {Smoke}!!} \textsc {Pick} \xrightarrow {\mathbf {Choose _i}!!} \textsc {Report}$$ for $$i \in \{1,2\}$$, and
- $$\textsc {Env} \xrightarrow {} \textsc {Ask} \xrightarrow {\mathbf {Smoke}??} \textsc {Pick} \xrightarrow {\mathbf {Choose _i}!!} \textsc {Report}$$ for $$i \in \{1,2\}$$.

It is clear that all the sending transitions $\mathbf {Smoke}!!,\mathbf {Choose _1}!!,\mathbf {Choose _2}!!$ appear only in $\mathcal {F}$. Furthermore, the corresponding broadcast-receive transitions satisfy the required conditions as follows:

- the transition $\textsc {Env} \xrightarrow {\mathbf {Smoke}??} \textsc {Idle}$ satisfies condition (2a) because the internal transition $\textsc {Env} \xrightarrow {} \textsc {Ask}$ exists in a path in $\mathcal {F}$.
- the transition $\textsc {Pick} \xrightarrow {\mathbf {Choose}??} \textsc {Idle}$ satisfies condition (2b) since all paths out of Idle are free (namely, the negotiation transition $\textsc {Idle} \xrightarrow {\mathbf {Reset}} \textsc {Env}$) and lead back to a path in $\mathcal {F}$.

Since Lemma 3 holds, the reachability of $s$ is synchronization-independent and the cutoff is 3.

**Checking the Cutoff Conditions.** Note that while the conditions in Lemma 3 seem complex, all our cutoff conditions can be checked on the process definition in polynomial time, making them well-suited for fully automatic verification.

## Applications and Evaluation

To evaluate our approach, we consider several distributed applications that use agreement protocols like consensus or leader election, and that can be modeled as well-behaved systems that satisfy one of our cutoff lemmas:

- Chubby [11]: A distributed lock service for coarse-grained synchronization with an elected leader node that handles client messages.
- Distributed Smoke Detector (SD): A sensor network application that elects a subset of processes, who have detected smoke, to report to the authorities.
- Smoke Detector with Reset (SDR): A variant of SD that uses a “reset” signal to resume monitoring for smoke, thereby requiring infinite rounds of agreement. (this was our motivating example in Fig. 1)
- Distributed Mobile Robotics (DMR): Based on an existing benchmark [18], where a set of robots successively coordinate to create a motion plan.
- Distributed Key-Value Store (KVS) modeling a key-value store á la Redis [48].
- Small Aircraft Transportation System (SATS): The landing protocol of SATS proposed by NASA [53]. SATS aims to increase access to small airports without control towers by allowing aircrafts to coordinate with each other to operate safely upon entering the airport airspace.
- SATS$^{++}$: A variant of the SATS protocol where all processes communicate explicitly to determine subsets of aircrafts to coordinate the landing with.

In addition, we provide an experimental evaluation, based on related work [37] in which a new model—the Choose model—that can be seen as a refinement of GSP, is proposed. The Choose model extends a standard model of distributed systems [2, 3] with a primitive that abstracts various types of distributed agreement protocols. The work further defines a mapping from the Choose model to GSP that establishes a simulation equivalence between the two models, enabling interchange of safety verification and cutoff results between the two models.

**Table 1.** Performance of parameterized verification based on our cutoffs.

Benchmark	States	Cutoff	Verification time(s)
Chubby	9	2	0.12
SD	5	3	0.28
SDR	5	3	0.13
DMR	8	3	0.16
KVS	18	3	3.06
SATS	24	5	3.83
SATS$^{++}$	26	5	17.1

**Fig. 3.**
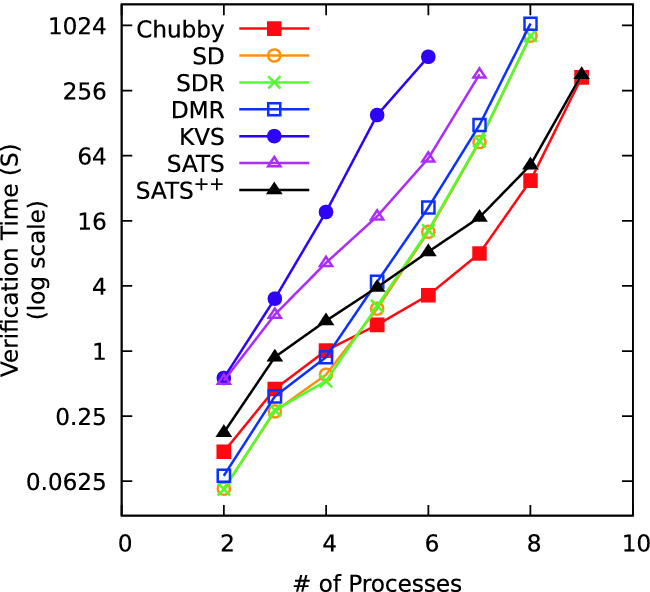
Verification time as a function of the number of processes.

To make use of the ease of encoding the above benchmarks in the Choose model and the ease of verification in the Choose model using off-the-shelf model checkers, we illustrate the effect of our cutoff results on efficiency of verification in the Choose model. For the benchmarks given above, Fig. 3 depicts the verification time as a function of the number of processes. Observe that verification time grows roughly exponentially with the number of processes. Moreover, verification for all the benchmarks timed out beyond 9 processes, for a timeout of 30 min. In contrast, in Table 1 all benchmarks have a cutoff of less than 6, and reasonable verification times.

## Related Work

Bodies of work that aim at automatically solving the parameterized verification problem (which is undecidable in the most general case [23, 54]) take a large variety of different approaches [1, 10, 13, 33, 35, 41, 43, 47, 56], in most cases without a focus on decidability. In the following we consider the approaches that target decidability, with models closely related to our GSP model.

**Models with Broadcasts and/or Global Guards.** We want to enable reasoning about distributed systems, abstracting complex building blocks like agreement protocols by primitives that satisfy *assume-guarantee* specifications. To support parameterized reasoning for systems with such abstractions, one needs a model with (i) conjunctive guards to model the assumptions, and (ii) forms of synchronization that are sufficiently general to model the guarantees of those building blocks, i.e., generalizations of broadcast communication.

Esparza et al. [28] present a decidability result for safety properties of broadcast protocols, but without global guards. Their result is also based on a reduction to WSTSs, but we showed that the WQO presented in their work (corresponding to the WQO $\preceq$ in Sect. 3.2) is not suitable for systems with guards. We note that our GSP model subsumes the model of Esparza et al., and that our cutoff results also apply to their model (which had no previous cutoff results).

Other existing models either are not sufficiently general [19, 20, 22], or support a combination of broadcasts and conjunctive guards without restrictions [21], which makes safety undecidable. This highlights the significance of our result: we manage to find a model with conjunctive guards and global synchronization such that safety remains decidable.

**Other Decidable Classes.** One way to obtain decidability is to restrict the generality of the parameterized verification problem in various ways. Most results in this direction consider a fully connected network (a clique), either with rendezvous communication [5, 32], local updates with global guards [6, 19], or variants of these [16]. Some communication primitives have also been considered in more complex networks, for example token passing [4, 12, 24], or broadcasts [17]. Decidability results for systems that are composed of identical components have recently been surveyed by Bloem et al. [9] as well as Espazra et al. [26]. Several bodies of work attempt to identify cutoff bounds for different classes of distributed systems. For example, cutoffs have been obtained for cache coherence protocols [20], guarded protocols [19, 21, 40], consensus protocols [44], and self-stabilizing systems [8]. None of these approaches are sufficiently general to tackle the types of distributed applications we address.

**Petri Nets and Vector Addition Systems.** Also closely related to the parameterized verification problems we consider is the body of work on Petri nets and vector addition systems, surveyed e.g. by Esparza and Nielsen [29] or Reisig [49]. While some types of communication can faithfully be expressed in these systems, global synchronization in general cannot.

## Conclusion

We introduced global synchronization protocols (GSP), a system model that generalizes many existing models supporting global synchronization such as broadcast synchronization, pairwise rendezvous, and asynchronous rendezvous. We identified sufficient conditions, summarized under our notion of well-behavedness, that ensure decidability of the parameterized verification problem even in the presence of global (conjunctive) transition guards. Finally, we investigated cutoffs for parameterized verification, and identified sufficient conditions under which small cutoffs exist.

In ongoing work, we are focusing on extensions of our cutoff results as well as a dedicated implementation of our decision procedure. In the near future, we plan to investigate sufficient conditions that enable support for the parameterized verification of liveness properties for GSPs, and intend to develop a domain-specific language for writing GSPs that are well-behaved by construction.

## Footnotes

1 Processes that do not satisfy the assumptions can easily be rewritten to satisfy them, e.g. by adding self-loops on any missing receive actions, and by renaming the actions of duplicate sending transitions (and adding corresponding receiving transitions).

2 For presentation clarity, we do not explicitly consider an environment process in our model. All of our results extend to the case with an explicit environment process; see the extended version [38] for a justification.

3 We show that $\trianglelefteq$ is a WQO by proving that every infinite sequence of global states $\mathbf {q}_1, \mathbf {q}_2, \ldots$ contains $\mathbf {q}_i, \mathbf {q}_j$ with $i <j$ and $\mathbf {q}_i \trianglelefteq \mathbf {q}_j$. To this end, consider an arbitrary infinite sequence $\overline{\mathbf {q}} = \mathbf {q}_1, \mathbf {q}_2, \ldots$. Then there is at least one set *S* of local states such that infinitely many $\mathbf {q}_i$ have $\mathsf {supp} (\mathbf {q}_i)=S$. Let $\overline{\mathbf {q}'}$ be the infinite subsequence of $\overline{\mathbf {q}}$ where all elements have $\mathsf {supp} (\mathbf {q}'_i)=S$. Since $\preceq$ is a WQO, there exist $\mathbf {q}'_i, \mathbf {q}'_j$ with $i<j$ and $\mathbf {q}'_i \preceq \mathbf {q}'_j$, and since $\mathsf {supp} (\mathbf {q}'_i)=\mathsf {supp} (\mathbf {q}'_j)=S$, we also get $\mathbf {q}'_i \trianglelefteq \mathbf {q}'_j$. Since $\mathbf {q}'_i = \mathbf {q}_k$ and $\mathbf {q}'_j = \mathbf {q}_l$ for some $k < l$, we get $\mathbf {q}_k \trianglelefteq \mathbf {q}_l$ for $k < l$, and thus $\trianglelefteq$ is a WQO.
